# Anti proliferative and apoptotic effects on pancreatic cancer cell lines indicate new roles for ANGPTL8 (Betatrophin)

**DOI:** 10.1590/1678-4685-GMB-2019-0196

**Published:** 2020-07-31

**Authors:** Fatemeh Taherkhani, Kamran Mousavi Hosseini, Sanaz Zebardast, Koorosh Goodarzvand Chegini, Nematollah Gheibi

**Affiliations:** 1 Iranian Blood Transfusion Organization, Research Center, Tehran, Iran.; 2 Cellular and Molecular Research Center, Research Institute for Prevention of Non-Communicable Diseases, Qazvin University of Medical Sciences, Qazvin, Iran.

**Keywords:** Betatrophin, Wnt, WIF1, Bcl2, pancreatic cancer

## Abstract

Despite considerable advances, the treatment of pancreatic cancer (PC) still requires much effort. Unusual regulation of the Wnt and apoptotic signaling pathways is widespread in cancer incidence. For instance, the *WIF1* (Wnt inhibitory factor 1) gene is down-regulated in many cancers. The purpose of this study was to determine the effects of recombinant Betatrophin, a recently discovered hormone, on MiaPaca-II and *Panc-1* pancreatic cell lines. Various concentrations of Betatrophin were added to MiaPaca-II and *Panc-1* pancreatic cell lines during periods of 24 , 48, and 72 h. The MTT assay was applied to investigate cell proliferation after treatment. The rate of apoptotic cells was assessed using double-staining flow cytometry, and the expression levels of the *WIF1* gene and Bcl2 protein was observed by real-time PCR and western blotting, respectively. The findings of this study suggest that Betatrophin has an anti-proliferative effect on both MiaPaca-II and Panc-1 cell lines, in line with the up-regulation of *WIF1* as a tumor suppressor gene. Moreover, the induction of apoptosis by ANGPTL8 occurred by the down-regulation of Bcl2. Thus, Betatrophin can be proposed as a potential therapeutic drug for treating pancreatic cancer.

## Introduction

Pancreatic cancer is one of the most fatal types of cancer. Since this cancer does not have an early prognosis, just under 20% of the patients live for more than one year after diagnosis (Bailey *et al.*, 2016; Waddell *et al.*, 2015). There are some factors that lead to the reduced survival rate of this disease. One is the difficulty in diagnosis during the early stages of the disease. Moreover, tumors progress rapidly while having few specific associated symptoms, and different pancreatic cancers show different responses to related drugs. Although there has been a progression towards figuring out the histological characteristics and molecular mechanisms underlying cancer development, studies showing favorable responses to available drugs continue to be rare. As a result, the survival chances of patients have not significantly improved (Siegel *et al.*, 2013; Sahmani *et al.*, 2016). A major obstacle for following a better treatment plan has been the heterogeneity of these cancers. This is because of the vast amount of somatic mutations acquired during the development of a tumor, and the different consequences of these mutations on cell signaling pathways (Sousa *et al.*, 2013; Hidalgo *et al.*, 2015).

The Wnt signaling pathway is responsible for controlling progress such as embryonic development, cell proliferation, polarization, cell fate, and the process of renewing in stem cells (Kudo, 2010). It has been indicated that this pathway has a decisive role in numerous malignancies, including breast cancer (Geyer *et al.*, 2011), colon cancer (Vermeulen *et al.*, 2010), leukemia (Luis *et al.*, 2012), gastric cancer (Yong *et al.*, 2016), esophageal cancer (Zhang *et al.*, 2016a), and HCC 7 (Fatima *et al.*, 2012), for instance. A role for the Wnt signaling pathway has also been reported in pancreas development (Hebrok, 2003; Dessimoz *et al.*, 2005; Murtaugh *et al.*, 2005, Papadopoulou and Edlund, 2005; Heiser *et al.*, 2006). During early pancreatic development, incorrect activation of Wnt signaling causes imperfect development of this organ (Heller *et al.*, 2002; Heiser *et al.*, 2006).

Expression of the *Wnt inhibitory factor 1* (*WIF1*) gene prevents receptor interactions and induces β-catenin degradation by binding directly to Wnt ligands situating outside the cell. Down-regulation of *WIF1* has been reported prostate, breast, lung, and bladder cancers. Silencing of the *WIF1* gene due to promoter hyper-methylation has been revealed in gastrointestinal, lung and bladder cancers (Taniguchi *et al.*, 2005; Urakami *et al.*, 2006; Yoshino *et al.*, 2009; Rahmani *et al.*, 2017). It has also been observed that stimulation of *WIF1* activity in cancer cells allowed to treat some malignant cancers (Ng *et al.*, 2014). Although, activation of the Wnt pathway seems to be involved in pancreatic cancer (Wang *et al.*, 2009), the expression and precise function of *WIF1* in pancreatic cancer progression have not been determined so far.

Apoptosis is a vital biological process that controls homeostasis and the dynamic balance between cell proliferation and cell death (Tabas and Ron, 2011), and the *Bcl2* gene family with about 25 members plays a key role in the regulation of the intrinsic or mitochondrial apoptotic pathway. However, for the understanding of the apoptotic effect in pancreatic cancer cell lines clarification is needed on the regulation of the pro-apoptotic gene *Bax* and the anti-apoptotic gene *Bcl2*, which play a significant role in the intrinsic pathway of apoptosis.

Betatrophin, also known as angiopoietin-like protein (ANGPTL8), is a recently identified circulating protein that is mostly produced in the liver and adipose tissues. The human *Betatrophin* gene has four exons encoding a protein with 198 amino acids. In various studies, its role has been determined in glucose and lipid metabolism, metabolic diseases (Crujeiras *et al.*, 2016), polycystic ovary syndrome (PCOS) (Calan *et al.*, 2016), adriamycin cardiomyopathy (Chen *et al.*, 2016b), and renal dysfunction (Chen *et al.*, 2016a).

The focus of this study was to assess its effects on cell proliferation and apoptosis in the MiaPaca-II and Panc-1 as pancreatic cancer cell lines treated with different concentrations of Betatrophin. The effects of Betatrophin on Wnt and apoptosis signaling pathways was assessed by measuring the expression level of *WIF1* as a tumor suppressor gene by real-time PCR, and the expression of Bcl2 protein by western blot analysis.

## Material and Methods

### Cloning of *Betatrophin*

*Betatrophin* was cloned using the PET28 plasmid as vector for transformation of *E. coli* BL21 cells. The procedures of cloning and purification are described in our previous study (Gholami *et al.*, 2017).

### Cell culture

The human pancreatic cancer cell lines MiaPaca-II and Panc-1 were purchased from the Pasteur Institute of Iran and cultured in T-25 flasks (Jet Biofil Flask) using 4-6 mL of DMEM-high glucose medium containing 10% fetal bovine serum (Gibson,26140-079) and 1% antibiotic of penicillin–streptomycin (Gibson,15140-122). They were cultured at 37 °C under 5% CO_2_ atmosohere. When cells reached 80% confluency in the subculture, the overlying medium was removed, and the cells were washed two times with 1 mL PBS. After adding trypsin-EDTA 0.25% (Gibson, 25200-056), the cells were incubated for 2-3 min before adding 3 mL of DMEM medium to neutralize the trypsin effect. They were transferred to 15 mL Falcon tubes and centrifuged for 7 min at 1700 rpm. The supernatant medium was removed, new medium was added to the pelleted cells, and these were then transferred to incubation flasks. For freezing, 1 mL of freezing medium (5-10% DMSO with 90-95% FBS) was used.

### Cell toxicity assessment by MTT assay

Cell viability was tested using the colorimetric [3-(4, 5-dimethylthiazol-2yl-)-2,5-diphenyl tetrazolium bromide] tetrazolium reduction assay (MTT). The MiaPaca-II and Panc-1 cell lines were seeded at a concentration of 510^3^ cells/well and incubated for 24 h. After termination of the incubation period, the cells were exposed to 200 μL of increasing concentrations of Betatrophin (5, 10, 20, 40, 50, 75, 100, 125, 150 pg/mL) for 24, 48 and 72 h. After 24 h, the overlying medium was removed, and 180 μL of new medium was added to each well. At the end of the treatment period, the medium as removed and 20 μL of MTT (Sigma, Germany) was added to each well, followed by incubation for 4 h at 37 °C in the dark. After incubation, the MTT solution was removed and replaced with 200 μL DMSO. The cells were then incubated for 10 min in a shaking incubator. Glycine buffer was added and absorbance was evaluated at 570 nm in an ELISA plate reader. The assay was performed in triplicate.

### Apoptotis assay via flow cytometry

Assessment of apoptotic cells was done using the annexin V/PI double-staining flow cytometry detection kit (Biolegend). After culturing MiaPaca-II and Panc-1 cell lines and treating them with 150M concentrations of Betatrophin for 24, 48 and 72 h, the cells were trypsinized and collected by centrifugation (350 × *g*, 5min). Annexin-V and PI conjugated with FITC were added to the cells, and they were incubated at room temperature for 15 min. The fluorescence distribution was recorded in a two-color dot blot analysis, and the percentage of fluorescent cells was determined.

### RNA extraction and cDNA synthesis

Total RNA of treated and untreated MiaPaca-II and Panc-1 cells was extracted using the BioFACT kit (Cat.No.RP101-050/RP101-100; South Korea) according to the manufacturer’s guidelines. Quantity and purity of the extracted RNA was assessed in a Nanodrop spectrophotometer at the wavelength range of 90 – 320 nm. cDNA was synthesized using the BioFACT kit (Cat.No.BR631-096) according to the manufacturer’s instruction. The quality of the synthesized cDNA was assessed using Nanodrop spectrophotometry and gel electrophoresis.

### Real-time PCR expression analysis of the *WIF1* gene

The effects of Betatrophin on *WIF1* gene expression were assessed by real-time PCR. Primers were obtained from Macrogen (South Korea). Their sequences were: for GAPDH 5- CAA TGACCCCTTCATTGACC -3 and 5-TGGAAGATGGTGATGGGATT-3; and for WIF-1 5-CC GAAATGGAGGCTTTTGTA-3 and 5-TGGTTGAG CAGTTTGCTTTG-3. Amplification was conducted in 20 μL of SYBR Green PCR Master Mix (qPCRBIO Syber-Green Mix Separate-Rox (NGS2X)) under the following conditions: initial denaturation at 95 °C for 15 min, 40 cycles at 95 °C for 20 s, annealing at 60 °C for the 30 s and extension at 72 °C for 30 s. Data were analyzed by the Pfaffl method, and the graphs were drawn by REST software 2009.

### Expression of of *Bcl2* by western blot analysis

MiPaca-II and Panc-1 cells were seeded and incubated for 24 h and then treated with a 150 μM solution of Betatrophin. Total protein extracts of the two cell lines were produced after 24, 48, and 72 h of treatment times using cell lysis buffer. Equal quantities of protein (50 μg) were resolved by SDS-PAGE and gels were transferred to nitrocellulose membranes. Non-specific binding sites were blocked by incubation in blocking buffer (PBS containing 0.1% Tween 20 and 5% non-fat dry milk) for 24 h at 4 °C. After washing the membranes twice, they were immunoblotted using the anti-β actin, anti-Bcl-2 primary antibodies at 4 °C overnight and then incubated with the corresponding HRP conjugated secondary antibodies for 1 h at room temperature. Western blot bands were detected using an enhanced chemiluminescence (ECL) detection system (GeneGnome XRQ - Chemiluminescence imaging). Band intensities were quantified and normalized to β-actin using the NIH ImageJ software.

## Results

### Viability assay by MTT

**Figure 1:**
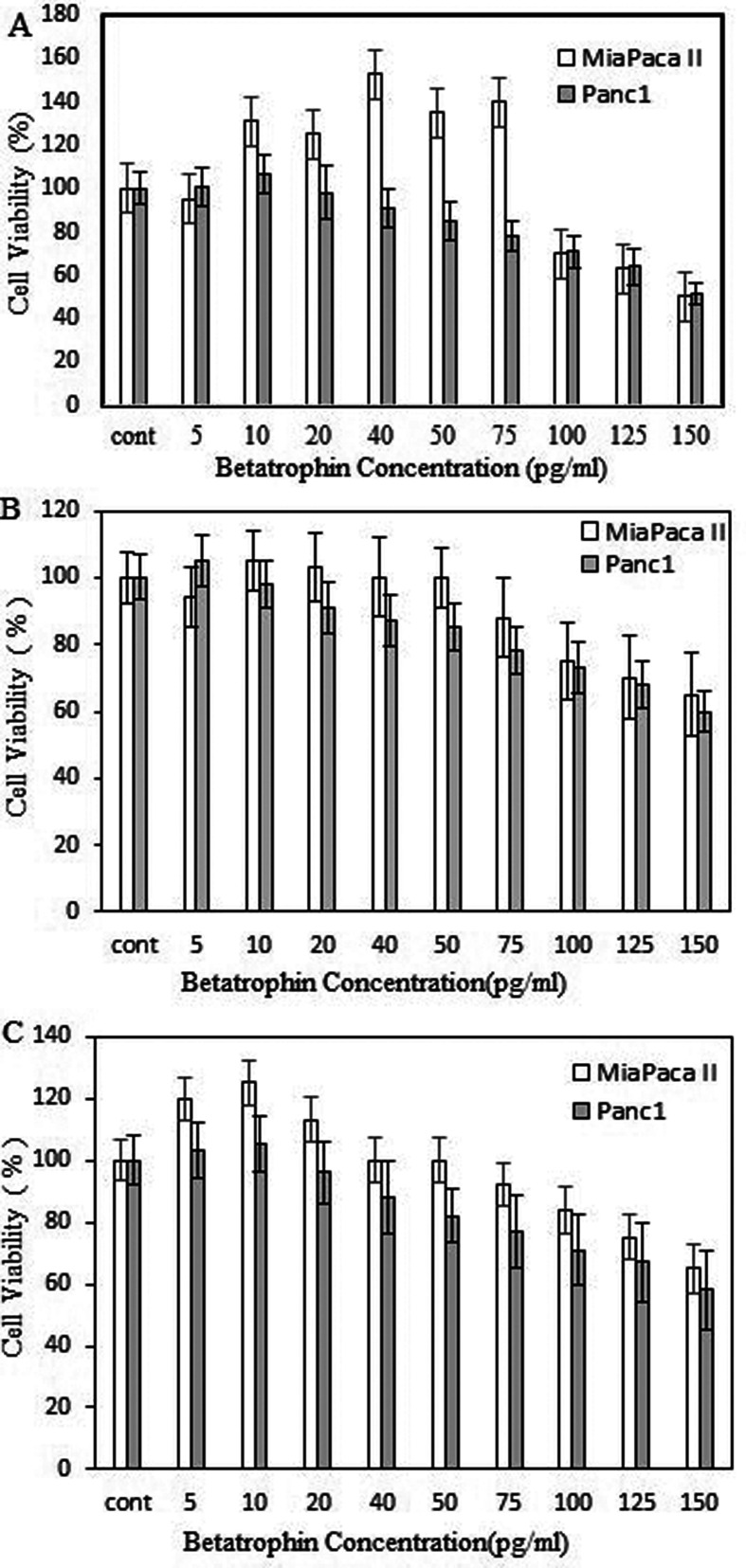
Cytotoxic effect of Betatrophin concentrations on MiaPaca II and Panc-1 human pancreatic cancer cell lines. The percentage of cell viability was measured by MTT assay at 24 h (A), 48 h (B), and 72h (C).

MiaPaca-II and Panc-1 cell lines were treated with several concentrations of Betatrophin and their viability was assessed by MTT assay. As shown in Figure 1A-C, the cell viability of both treated cell lines was decreased at the concentrations of > 75 pg/mL at all treatment times (24, 48, and 72 h).

### Apoptotic effect of Betatrophin

**Figure 2:**
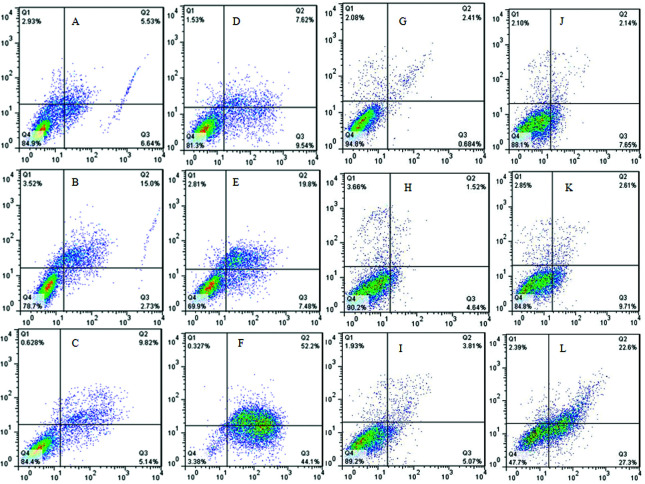
Effect of different concentration of Betatrophin on apoptosis. Annexin V/PI double-staining flow cytometric assay of MiaPaca-II (A-F) and Panc-1 (G-L) pancreatic cancer cell lines after treatment with 150 pg/mL Betatrophin on. (A and G) Untreated cells at 24 h; (B and H) untreated cells at 48 h; (C and I) untreated cells at 72 h; (D and J) treated cells at 24 h. (E and K) treated cells at 4 8h; (F and L) treated cells after 72 h.

**Figure 3:**
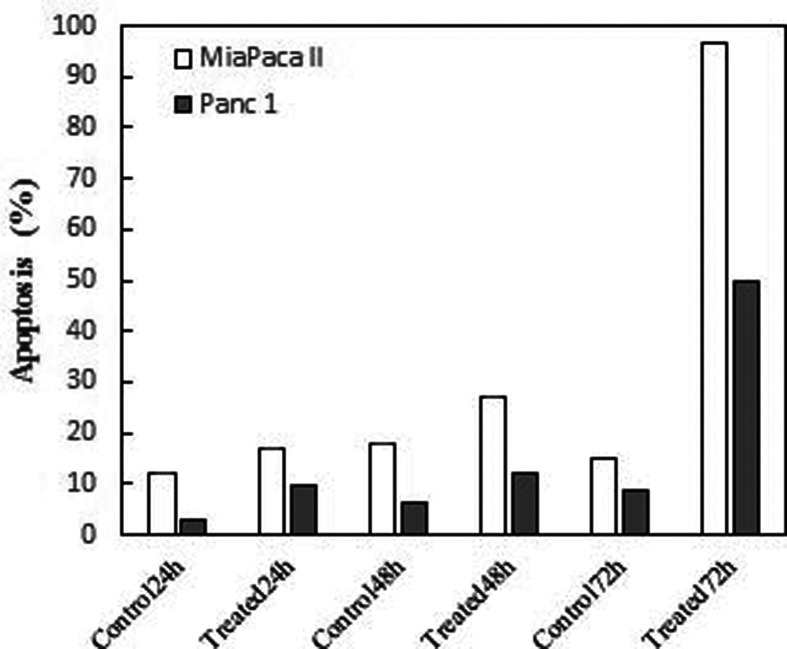
Percentage of apoptosis induced by 150 pg/mL betatrophin on MiaPaca II and Panc-1 pancreatic cancer cell lines at 24 h, 48 h, and 72h treatment times.

The apoptotic effect of Betatrophin on MiaPaca-II and Panc-1 cell lines was assessed by double-staining Annexin-V/PI flow cytometric analysis. As illustrated in Figure 2A-L the dot-plot data for 24, 48, and 72 h treatment times show the occurrence of apoptosis for both cell lines treated with 150 pg/mL of Betatrophin. The overall percentage values of Betatrophin that induced apoptosis in early and late apoptotic cell populations at the three treatment times are shown in Figure 3.

### Expression changes of *WIF1*

**Figure 4:**
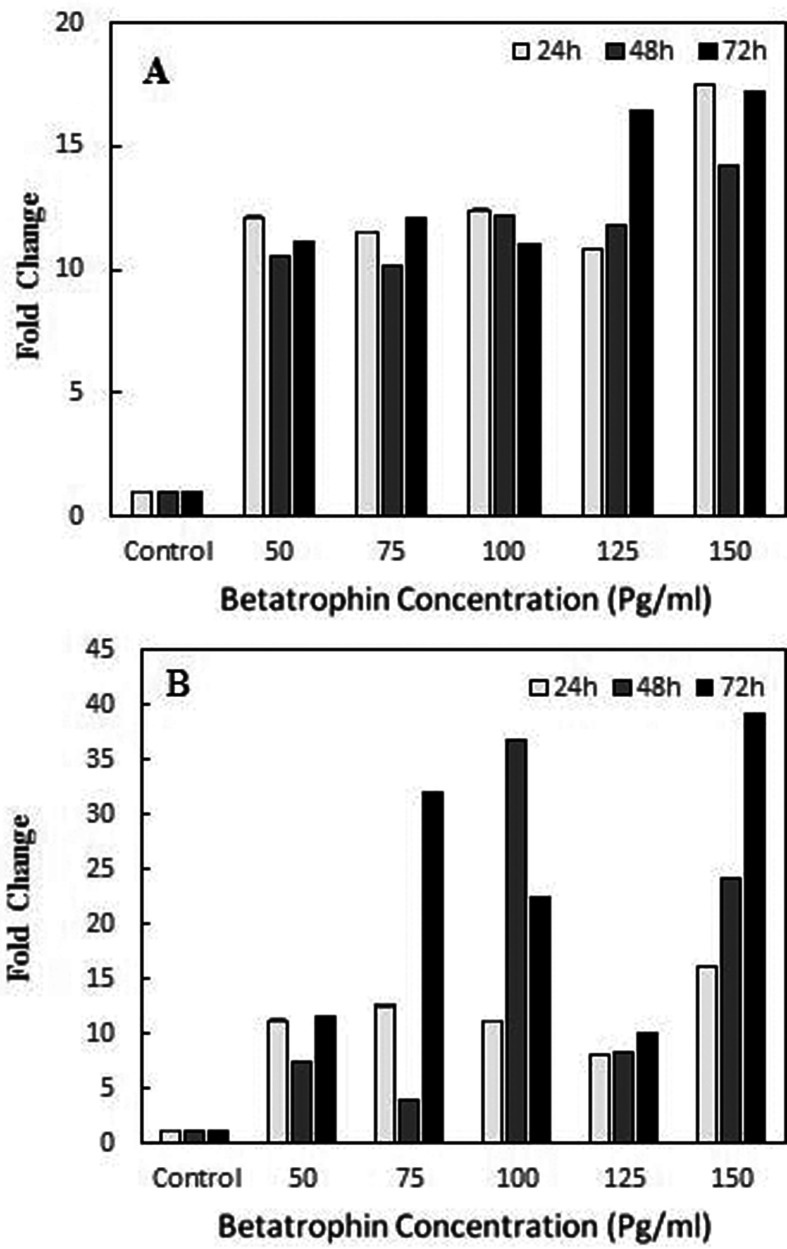
Effect of different concentration of Betatrophin on the expression level of WIF1. The effects was asseded in MiaPaca-II (**A**) and Panc-1 (**B**) pancreatic cancer cell lines after 24 h, 48 h and 72 h treatment times.

Expression of the *WIF1* gene was compared in both treated and untreated cells. GAPDH was considered as an internal control for normalization. As shown in Figure 4A and B, the expression of *WIF1* in both cell lines increased in comparison to control in a concentration-dependent manner.

### Changes Bcl-2 protein levels

**Figure 5:**
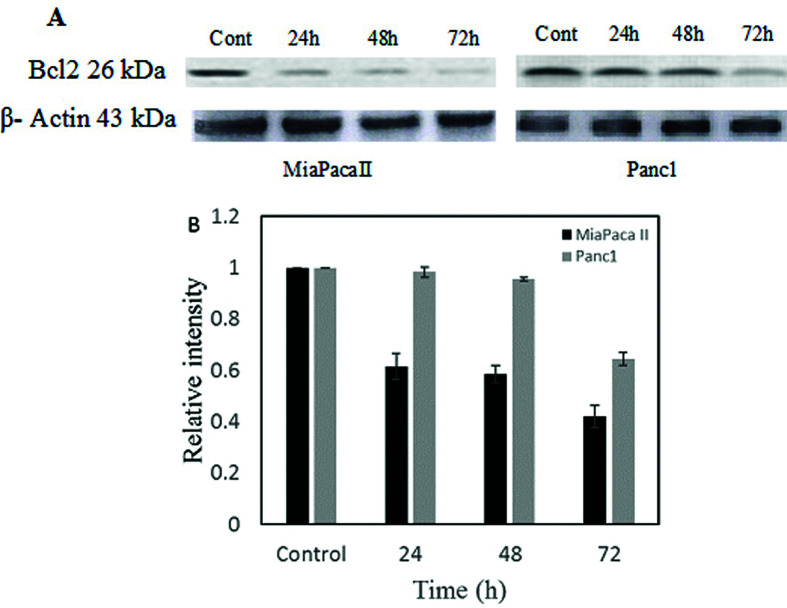
Detection of Bcl2 protein by western blot analysis. (A) Bcl2 expression in MiaPacaII and Panc-1 panceratic cells at 24 h, 48 h and 72 h treatment times. β-Actin was used as loading control. (B) Bcl2 expression levels in untreated and treated cells were quantified by Image-J software and normalized to band intensity of β-actin.

For further analysis of apoptosis in Betatrophin-treated MiaPaca-II and Panc-1 cells, Bcl2 expression was assessed, as this is the main apoptosis-related gene. The protein level of Bcl2 was measured before and after treatment with 150 M Betatrophin at 24, 48 ,and 72 h. We found that the level of anti-apoptotic protein Bcl-2 was dramatically reduced in both pancreatic cell lines (Figure 5).

## Discussion

The results of the current study showed that Betatrophin induces anti-proliferative and apoptotic effects on the two pancreatic cancer cell lines MiaPaca-II and Panc-1. Inhibition of the Wnt signaling pathway was induced by the up-regulation of *WIF1* as a tumor-suppressor gene. Betatrophin-induced apoptosis was investigated through the down-regulation of Bcl2 as an anti-apoptotic protein.

Numerous previous studies had reported that reducing the Wnt pathway targets cancer stem cells (Kim *et al.*, 2018) and induces instant and substantial death in several cancer cell lines, including lung, breast, mesothelioma, and sarcoma, which all overexpress *Wnt-1* (He *et al.*, 2004). Inhibition of the Wnt signaling pathway resulted in suppression of cancer metastasis (Cao *et al.*, 2017) and prevents the proliferation of cancer cells (Choi *et al.*, 2010). Other findings suggest that this signaling pathwayis crucial inpancreatic cancerand may be a target for drug therapy (Garg *et al.*, 2017, Dehghanifard *et al*., 2018). Poorly regulated Wnt/β-catenin signaling has also been shown to be involved in the chemo-resistance of pancreatic cancer (Cui *et al.*, 2012). A study done in 2017 showed that the microRNA-195 inhibits the spreading of pancreatic cancer cells by limiting the fatty acid synthase/Wnt signaling pathway. This study suggested that microRNA-195 can act as a tumor suppressor in the expansion of pancreatic cancer (Xu *et al.*, 2017). A monoclonal antibody (OMP-18R5) that inhibits the Wnt signaling pathway in numerous tumors, including pancreatic ones, by targeting Frizzled receptors showed conspicuous synergy when combined with gemcitabine (Gurney *et al.*, 2012). It was also demonstrated that Wnt-inhibitors, such as ethacrynic acid (EA), ciclopirox olamin (CIC), piroctone olamine (PO), and griseofulvin (GF) reduce the viability of a murine and a human pancreatic cell line (Wall and Schmidt-Wolf, 2014). As shown here (Figure 1) Betatrophin-recombinant protein reduced cell viability of the MiaPaca-II and Panc-1 cell lines in a dose-dependent manner. The expression levels of *WIF1* demonstrated regulatory effects of this tumor suppressor gene on the Wnt signaling pathway and thus, the anti-proliferation effect of Betatrophin (Figure 2).

ANGPTL proteins became attractive as prognostic or predictive indicators and as a new treatments for curing cancers (Carbone *et al.*, 2018). ANGPTL8 (Betatrophin) ameliorates the inability of insulin in increasing glucose via the Akt-GSK3β or Akt-FoxO1 pathway in HepG2 cells (Guo *et al.*, 2016). It has been reported that this protein activates the ERK signal transduction pathway in hepatocytes, pancreatic β-cells, and adipocytes, causing down-regulating adipose triglyceride lipase (Zhang *et al.*, 2016b). Betatrophin probably makes use of the macrophage receptor for regulating lipid/triglyceride metabolism, and the neuronal receptor mediating the signaling to pancreatic beta cells via nerves(Yi *et al.*, 2014). It seems that increased levels of Betatrophin in serum in pancreatic cancer-associated diabetes (Susanto *et al.*, 2016) may be a protective mechanism limiting the proliferation of cancer cells.

Applying flow cytometry to evaluate the apoptotic role of Betatrophin on MiaPaca-II and Panc-1 cell (Figures 3 and 4) showed that it increased the overall percentage of early and late apoptosis compared to control untreated cells, especially after 72 hours treatment time. Apoptosis related genes, including *Bcl2*, *Bcl-xL*, and caspase-3 can be regulated by NF-κB, thus inhibiting the apoptosis of pancreatic cells (Banerjee *et al.*, 2005; Kunnumakkara *et al.*, 2007). We found a significant decrease in the level of Bcl2 protein (Figure 5) indicating that induction of apoptosis by ANGPTL8 (Betatrophin) was achieved by down-regulation this protein. Higher expression of *Bcl2* in cancer cells is known to lead to tumor progression by preventing apoptosis (Florou *et al.*, 2013). Since the mitochondrial membrane potential is preserved by *Bcl2*, and its overexpressing causes a less pronounced decrease of mitochondrial depolarization, it is reasonable to assume that mitochondrial fission and fusion occur by reducing the level of *Bcl2* in ANGPTL8-treated cells.

In the current study, higher expression of *WIF1* was observed after treatment with Betatrophin in the MiaPaca-II and Panc-1 pancreatic cancer cell lines. Decreased expression of *WIF1* was reported in many cancers, such as gastrointestinal tract, kidney, glioblastoma, osteosarcoma, lung, pituitary, bladder, and oral cavity (Mazieres *et al.*, 2004; Taniguchi *et al.*, 2005; Urakami *et al.*, 2006; Elston *et al.*, 2007; Kawakami *et al.*, 2009; Rubin *et al.*, 2010; Lambiv *et al.*, 2011; Paluszczak *et al.*, 2015). When re-expressed, *WIF1* can down-regulate the Wnt pathway and prevent cancer cell growth (Gao *et al.*, 2009; Kawakami *et al.*, 2009; Yee *et al.*, 2010; Hirata *et al.*, 2011; Ramachandran *et al.*, 2012; Jiang *et al.*, 2016). In line with our study, down-regulation of the *WIF1* gene was observed in pancreatic cancer tissues, and this was attributed to hypermethylation of the *WIF1* promoter region. Treatment with the demethylating agent 5-aza-20-deoxycytidine (5-aza-dC) re-established *WIF1* expression in cancer cell lines. It was suggested that managing the Wnt pathway would be a probable target for treatment and/or prevention of gastrointestinal cancers like pancreatic cancer (Taniguchi *et al.*, 2005; Azad *et al.*, 2013). Furthermore, in pancreaticductal adenocarcinoma (PDA), HOX transcript antisense intergenic RNA (HOTAIR) regulates theexpressionof *WIF1*, affecting the radiosensitivity ofpancreaticductal adenocarcinoma (Jiang *et al.*, 2016).

Taken together, it is plausible to say that Betatrophin has an anticancer effect on the pancreatic cancer cell lines used in this study, MiaPaca-II and Panc-1, by inhibiting cell growth and increasing *WIF1* gene expression, which subsequently reduces Wnt signaling as a decisive pathway in proliferation. Also, its anti-pancreatic cancer effect was shown by its induction of apoptosis and down-regulation of Bcl-2 as an anti-apoptotic protein.
